# Effects of Bacterial Secondary Symbionts on Host Feeding by *Aphelinus abdominalis*: Implications for the Biological Control of *Sitobion avenae*

**DOI:** 10.3390/insects17070744

**Published:** 2026-07-21

**Authors:** Sajjad Ali, Qaiser Shakeel, Muhammad Shahid Rizwan, Zaibun-Nisa Memon, Muhammad Yasin, Muhammad Irfan Ullah, Aristidis Matsoukis, Domenico Prisa, Aftab Jamal, Muhammad Farhan Saeed

**Affiliations:** 1 Department of Entomology, The Islamia University of Bahawalpur, Bahawalpur 63100, Pakistan; yasin_1876@yahoo.com; 2 Department of Crop Sciences, Georg-August University, 37073 Goettingen, Germany; 3 Cholistan Institute of Desert Studies, The Islamia University of Bahawalpur, Bahawalpur 63100, Pakistan; qaiser.shakeel@iub.edu.pk (Q.S.); shahid.rizwan@iub.edu.pk (M.S.R.); 4 Department of Zoology, Shah Abdul Latif University, Khairpur Mirs 66020, Pakistan; zaib.nisa@salu.edu.pk; 5 Department of Entomology, University of Sargodha, Sargodha 40100, Pakistan; muhammad.irfanullah@uos.edu.pk; 6 Department of Crop Science, School of Plant Sciences, Agricultural University of Athens, 75 Iera Odos St., 11855 Athens, Greece; 7 Research Centre for Vegetable and Ornamental Crops, Consiglio per la Ricerca in Agricoltura e l’Analisi dell’Economia Agraria (CREA), Via Dei Fiori 8, 51012 Pescia, Italy; domenico.prisa@crea.gov.it; 8 Department of Soil and Environmental Sciences, Faculty of Crop Production Sciences, The University of Agriculture, Peshawar 25130, Pakistan; aftabses98@gmail.com; 9 Department of Environmental Sciences, COMSATS University Islamabad, Vehari Campus, Vehari 61100, Pakistan; farhansaeed@cuivehari.edu.pk

**Keywords:** bacterial symbionts, *Sitobion avenae*, *Aphelinus abdominalis*, host densities, host feeding, biological control

## Abstract

*Aphelinus abdominalis* (Dalman) is a generalist aphid parasitoid that attacks a wide range of aphid species and, in addition to parasitism, also exhibits host-feeding behavior. A wheat aphid, *Sitobion avenae* (F.), frequently harbors bacterial secondary symbionts (BSS), i.e., *Hamiltonella defensa* and *Regiella insecticola*, known to provide protection against natural enemies. Parasitoids can discriminate among potential hosts based on symbiont infection status. Genetically identical clones of wheat aphid, either harboring or free of BSS, were established. The influence of BSS on host feeding activity and host selection of *A. abdominalis* was examined using both no-choice and choice assays with different host densities. BSS impact on adult body size of *A. abdominalis* within aphid hosts was also evaluated. Aphids harboring BSS showed lesser mortality caused by *A. abdominalis* host feeding under both no-choice and choice assays across all tested densities, but the host feeding activity of *A. abdominalis* increased with rising host density. Accordingly, mortality of *S. avenae* showed a significant upward trend as aphid density increased in both experimental setups. A notable finding was the preference of *A. abdominalis* for killing BSS-free aphids in choice assays. In the case of egg-laying, in BSS-infected aphids, the parasitoid’s body size within the aphids was negatively affected. No evidence was detected to support the horizontal transfer of BSS from *S. avenae* through *A. abdominalis*. These results suggest that within each aphid clone, the BSS presence enhanced resistance against parasitoids’ host feeding, whereas comparisons between aphid clones should be understood carefully because host genotype and symbiont species differed simultaneously. Overall, BSS and host genetic variation influence the parasitoid host-selection behavior, which poses serious implications for the effectiveness of biological control of the wheat aphid.

## Simple Summary

We explored how bacterial secondary symbionts (BSS) impact the interactions between the wheat aphid *Sitobion avenae* and its parasitoid *Aphelinus abdominalis*. Not only did we compare genetically identical aphid lines with and without their specific naturally occurring symbionts, but we also compared the two genetically distinct aphid clones without their BSS to evaluate the independent contribution of host genetic background. We then exposed them to the wasp at different densities and assessed host feeding, parasitoid preference, body size development, and symbiont transmission. Aphids harboring BSS suffered lower host-feeding mortality, while the parasitoids preferentially fed on BSS-free aphids. Parasitoid development was reduced in BSS-infected hosts, and no evidence of horizontal BSS transmission by the parasitoids was detected. These findings demonstrate that these symbiotic bacteria and host genetics can shift the balance in natural pest control, and it could help us design smarter crop protection.

## 1. Introduction

Symbiosis is defined as a close association between individuals of different species. An estimated 15–20% of insect species host symbiotic microorganisms, typically categorized as primary or secondary symbionts. These associations enhance the ability of insects to exploit diverse ecological niches by increasing nutritional adaptability [1,2]. Virtually all aphid species maintain an obligate association with *Buchnera aphidicola*, their primary endosymbiont [3]. This symbiont supplements the aphid diet by synthesizing essential amino acids that are lacking in phloem sap, thereby supporting host survival and fitness [4].

In addition to this primary partnership, aphids commonly harbor a range of BSS [5,6]. In aphids, BSS are well known for conferring resistance against both biotic stressors (e.g., natural enemies) and abiotic stressors (e.g., temperature extremes) [7,8,9,10]. They may also influence host plant specialization and aphid body coloration [11,12]. When providing partial resistance against parasitoids, BSS can suppress parasitoid larval growth and prolong developmental time within parasitized hosts, potentially reducing parasitoid survival [13,14]. These effects have primarily been demonstrated for parasitoid development following oviposition. To our knowledge, the influence of BSS on parasitoid host-feeding behavior has not been investigated, which is representing an important knowledge gap that the present study aims to address. BSS are typically transmitted vertically from mother to offspring through host reproductive processes [15]. However, both interspecific and intraspecific horizontal transmission can occur, albeit at low frequencies [15,16,17,18]. Thus, BSS represent important evolutionary partners that shape host adaptation and ecological interactions [6,19].

The English grain aphid, *Sitobion avenae* (F.), is one of the major pests of cereal crops worldwide, particularly in temperate regions [20,21]. It causes both direct and indirect damage by rapidly establishing large populations within a short period [22], resulting in significant yield losses in wheat [23,24]. Farmers are encouraged not to rely on insecticide use and adopt alternative ways to mitigate the yield losses [25]. Among the BSS associated with *S. avenae*, *Hamiltonella defensa* and *Regiella insecticola* are among the most commonly reported species, which were frequently reported for host protection against parasitoids in other aphid species [13,14,26].

Parasitoid-host interaction plays a central role in structuring trophic systems by regulating insect populations [27]. In addition to parasitism, many adult hymenopteran parasitoids engage in host feeding, which contributes substantially to host mortality [28,29,30]. Host feeding enhances parasitoid longevity and fecundity [28,31,32]. Non-reproductive host killing through host feeding is, therefore, considered an important trait that can increase the effectiveness of biological control agents [33], particularly within integrated pest management (IPM) frameworks.

Host feeding and killing generally increase with host density, although the proportion of hosts killed may decline as density increases [34]. Thus, host density is a key determinant of parasitoid foraging behavior and resource utilization. Parasitoids can detect the BSS presence in aphid hosts [35] and may also respond to genetic or clonal variations within the host populations [36,37]. *Aphelinus abdominalis* (Dalman) is an effective biological control agent against several aphid species. It exhibits both parasitism and host-feeding behavior, enabling it to suppress aphid populations through these two distinct mechanisms [30].

The role of BSS in parasitoid-aphid interactions has been extensively studied in pea aphids and, to a lesser extent, in bean aphids, primarily focusing on *Aphidius* species [13,38,39]. However, most studies have concentrated on parasitism, with limited attention to host-feeding behavior in relation to BSS infection status and host genetic variation under varying host densities. Moreover, interactions involving *S. avenae* and *A. abdominalis* remain poorly characterized in this context. To address this gap, we investigated the interactions among *S. avenae*, its BSS (*H. defensa* and *R. insecticola*), and *A. abdominalis*. Our experimental system contained two naturally occurring *S. avenae* clones differing in genetic background and associated BSS. Within each clone, genetically identical aphid lines, with and without BSS, were compared to evaluate the effects of BSS presence. Because BSS identity and host genotype differed simultaneously between clones, these assessments were interpreted carefully. This experimental design allowed us to study the influence of BSS on host feeding, parasitoid host preference, density-dependent host feeding, parasitoid body size, and the possible horizontal transmission of BSS by *A. abdominalis*. The findings could help improve biological control strategies to manage *S. avenae*.

## 2. Materials and Methods

### 2.1. Insect Cultures

*Sitobion avenae*: Two clones of *S. avenae* (hereafter referred to as clones 5 and 7) were used in this study. These clones were established from single parthenogenetic females collected from wheat fields in Giessen, Germany [21]. The clones differ in genotypic background and harbor distinct bacterial secondary symbionts (BSS): clone 5 carries *H. defensa* (HD), whereas clone 7 carries *R. insecticola* (RI) [40]. Both clones were maintained on wheat (*Triticum aestivum*) cultivar “Dekan” (KWS GmbH, Einbeck, Germany). Wheat plants were grown in 11 cm diameter plastic pots containing a 2:1 mixture of soil and sand. Each pot was enclosed in a transparent, ventilated plastic cylinder (10 cm diameter × 30 cm height). Aphids were transferred to fresh plants at a 15-day interval. Aphid cultures were maintained in climate chambers (WB 750 KFL; Mytron Bio & Solartechnik GmbH, Heiligenstadt, Germany) at 20 ± 1 °C, 70 ± 10% relative humidity, and a 16:8 h (L:D) photoperiod. Plants were irrigated on alternate days. Under these conditions, aphids reproduced parthenogenetically.

*Aphelinus abdominalis*: Commercial *A. abdominalis* were obtained from re-natur (GmbH, Ruhwinkel, Germany) as parasitized aphid mummies. Emerged adult parasitoids had no prior exposure to aphid hosts before the experiments. Upon arrival, newly emerged males and females were kept together for 24 h in aerated containers to allow mating and provided with a 50% (*v*/*v*) honey solution. Female parasitoids, approximately 24–48 h old, were starved for 12 h to standardize their physiological condition for host feeding before each bioassay.

### 2.2. Elimination of Bacterial Secondary Symbionts (BSS) from Aphid Clones

The antibiotic protocols used in this study were adopted from previously validated methods for the selective elimination of BSS and have been widely used in aphid-symbiont research. *Hamiltonella defensa* in clone 5 was selectively eliminated using an antibiotic cocktail consisting of ampicillin, cefotaxime, and gentamicin (each at 250 µg mL^−1^), administered via microinjection using fine glass capillaries [41]. The injection volume ranged from 0.1 to 0.2 µL mg^−1^ of aphid body weight. For selective removal of *R. insecticola* from clone 7, ampicillin was injected at a concentration of 1 µg mg^−1^ body weight [42]. Second-instar aphids were anesthetized with CO_2_ prior to injection. Following treatment, aphids were individually transferred to wheat plants and allowed to reproduce for 48 h. The resulting offspring were designated as the G1 generation. From each treated line, five G1 nymphs were randomly selected, reared to adulthood, and allowed to produce the G2 generation. G1 mothers were screened for BSS infection using diagnostic PCR. Only G2 offspring derived from BSS-negative G1 individuals were retained for further rearing. To minimize any transient physiological effects associated with antibiotic treatment, only G8 aphid lines were used for all experiments. Stable elimination of the target BSS was confirmed by diagnostic PCR across successive generations. Although the antibiotic protocols were adopted from previously published methods developed for the selective removal of *H. defensa* and *R. insecticola*, the abundance of the obligate symbiont *B. aphidicola* and other members of the aphid microbiome was not directly assessed following antibiotic treatment [43,44]. This procedure yielded four aphid lines: clone 5 (HD) with BSS (+5), clone 5 without BSS (−5), clone 7 (RI) with BSS (+7), and clone 7 without BSS (−7).

### 2.3. Diagnostic PCR for BSS Detection

Genomic DNA was extracted from aphid samples using the CTAB protocol [44,45]. Each diagnostic PCR assay included genomic DNA extracted from BSS-infected aphids as a positive biological control and DNA from confirmed BSS-free aphids as a negative biological control to verify the specificity of the amplification. The presence of *H. defensa* and *R. insecticola* was determined using diagnostic PCR targeting 16S rDNA fragments with the following primers: HDFn (5′-ATGAAGTCGCGAGACCAAA-3′), HDRn (5′-GCTTTCCCTCGCAGGTTC-3′), RIFn (5′-GAAGGCGGTAAGAGTAATATGC-3′), and RIRn (5′-CCCCGAAGGTTAAGCTACCTA-3′), respectively [44]. PCR conditions consisted of an initial denaturation at 94 °C for 3 min, followed by 30 cycles of 94 °C for 30 s, 60 °C for 40 s, and 72 °C for 90 s, with a final extension at 72 °C for 8 min. Reactions were performed in 25 µL volumes containing 1 µL DNA template, 0.32 µM of each primer, 2 mM MgCl_2_, 200 µM dNTPs, 1× Bioline buffer, and 0.25 U Taq DNA polymerase. PCR products were separated on 1.7% agarose gels stained with ethidium bromide. Selected amplicons were purified and sequenced (LGC Genomics GmbH, Berlin, Germany), and sequences were confirmed using BLAST (NCBI version 2.15) [44]. The assay was not intended to quantify symbiont titer; therefore, quantitative PCR (qPCR) was not performed.

### 2.4. Experiments

#### 2.4.1. Host Feeding Behavior of *A. abdominalis* in Response to BSS (No-Choice Assay)

Second- and third-instar nymphs of clones +5, +7, −5, and −7 were exposed to individual *A. abdominalis* females at densities of 6, 12, 18, 24, and 30 aphids per Petri dish (90 mm diameter) for 24 h. Each dish contained filter paper and washed, rooted wheat seedlings wrapped in moist cotton. Experiments were conducted at 20 ± 1 °C, 70 ± 10% RH, and a 16:8 h (L: D) photoperiod. Each treatment was replicated 16 times, with 16 control replicates lacking parasitoids. After 24 h, parasitoids were removed and preserved for DNA extraction. Host-feeding mortality was recorded for a 48 h period. Host-fed aphids were identified by their characteristic shrunken body appearance following hemolymph exuding and feeding [46]. The mortality recorded during the 48 h exposure period was attributed to host-feeding. Aphids surviving after 48 h were maintained until mummy formation to assess parasitoid development. Mummification data were not included in the host feeding mortality analyses. Emerging *A. abdominalis* adults were collected for hind tibia measurements and DNA analysis. Replicates in which parasitoids died or escaped were excluded and repeated.

#### 2.4.2. Host Preference Behavior in Response to BSS (Choice Assay)

For choice tests, equal numbers (50:50 ratio) of BSS-positive and BSS-negative aphids were placed together in a single Petri dish at the same density levels as above. To distinguish between treatments, the hind tarsus of one leg was clipped in one aphid group under a stereomicroscope. To avoid potential marking bias, the clipped treatment was alternated between BSS-positive and BSS-negative aphids across replicates. Experimental conditions, replication, and data collection procedures were identical to those described for no-choice tests.

#### 2.4.3. Host Preference in Response to Clonal Variation (Choice Assay)

To assess clonal effects, aphids from clones +5 and +7 (BSS-positive) and −5 and −7 (BSS-negative) were combined at a 50:50 ratio and exposed to *A. abdominalis* parasitoids under the same experimental conditions described above. These two aphid clones differed in both their genetic background and their original naturally occurring BSS associations. Clones were distinguished by clipping the hind tarsus of one group. To avoid potential marking bias, the clipping was alternated between the two aphid clones across the replicates. Data collection followed the same protocol as in previous experiments.

#### 2.4.4. Preference Index Calculation

To quantify host choice in the dual-choice assays, a standardized Preference Index (PI) was calculated for each experimental arena. For the dual-choice assay comparing BSS-free and BSS-infected aphids, the PI was calculated as

$$PI=\frac{K_{BSS-free}-K_{BSS-infected}}{K_{BSS-free}+K_{BSS-infected}}$$

where K (BSS-free) and K (BSS-infected) represent the numbers of BSS-free and BSS-infected aphids killed by the *A. abdominalis*, respectively.

For the dual-choice assay comparing aphid clones, the PI was calculated as

$$PI=\frac{K_{HD}-K_{RI}}{K_{HD}+K_{RI}}$$

where K HD and K RI represent the numbers of Clone 5 (HD) and Clone 7 (RI) aphids killed, respectively.

For ease of biological interpretation, both indices were expressed as percentages:

$$PI(\%)=100\times \frac{K_{1}-K_{2}}{K_{1}+K_{2}}$$

where K 1 and K 2 correspond to the two prey types being compared in each dual-choice assay.

The Preference Index ranges from −100 to +100, where 0 indicates equal predation on both prey types, positive values indicate preferential predation on the first prey category (BSS-free aphids or Clone 5), and negative values indicate preferential predation on the second prey category (BSS-infected aphids or Clone 7). The Preference Index was used solely as a descriptive measure of host choice, whereas statistical inference was based on grouped binomial generalized linear models [47,48].

#### 2.4.5. Effect of Bacterial Secondary Symbionts (BSS) on Adult Parasitoid Body Size

The hind tibia lengths (µm) of emerged *A. abdominalis* adults from mummified aphids were measured as an estimation of adult body size, following standard practice [49]. Measurements were taken under a stereomicroscope (Stemi 2000-C, Carl Zeiss, Oberkochen, Germany) equipped with a calibrated scale. At least 30 individuals per treatment (clones +5, −5, +7, −7) were measured.

#### 2.4.6. Transmission of BSS via *Aphelinus abdominalis*

To assess potential transmission of BSS, total DNA was extracted from female parasitoids recovered after 24 h from no-choice assays, as well as from *A. abdominalis* adults emerging from aphid mummies. Diagnostic PCR was performed using the same primers and amplification conditions described in Section 2.3. Parasitoids recovered from BSS-infected aphid lines (+5 and +7) served as positive controls, whereas parasitoids recovered from BSS-free aphid lines (−5 and −7) served as negative controls. The diagnostic PCR assay was used as a qualitative method to detect the presence or absence of BSS DNA in individual parasitoids and was not intended to quantify symbiont titer. A total of 48 parasitoids were collected after exposure, and 30 parasitoids emerged from mummies per treatment were analyzed.

### 2.5. Data Analysis

Mortality data from the no-choice assays were analyzed as grouped binomial responses using generalized linear models (GLMs) with a logit link. Aphid clone (HD and RI), bacterial secondary symbiont (BSS) status (BSS-free and BSS-infected), and host density (6, 12, 18, 24, and 30 aphids) were included as fixed effects. An initial generalized linear mixed model (GLMM) including replicate as a random effect was fitted, but because the random-effect variance was negligible, the final analyses were performed using binomial GLMs. Model simplification was based on likelihood-ratio tests, and the significance of explanatory variables was assessed using Type III likelihood-ratio (LR) χ^2^ tests. Adjusted probabilities were estimated using estimated marginal means (EMMs).

For the dual-choice assays, host choice was analyzed using grouped binomial GLMs. In the first assay, the numbers of BSS-free and BSS-infected aphids killed were analyzed using aphid clone and host density as explanatory variables. In the second assay, the numbers of Clone 5 (HD) and Clone 7 (RI) aphids killed were analyzed using BSS status and host density as explanatory variables. For both assays, non-significant interaction terms were removed during model simplification, and the overall probability of predator attack on each prey type was estimated from the final models and compared with the null expectation of equal allocation (0.50).

The Preference Index (PI) values were used solely as descriptive measures of host choice, whereas statistical inference was based on the grouped binomial GLMs. Model assumptions were evaluated using simulation-based residual diagnostics. Because residuals indicated under-dispersion but no evidence of overdispersion or influential outliers, standard binomial GLMs were retained as the primary analytical approach, with quasibinomial models fitted as sensitivity analyses.

#### 2.5.1. Functional Response Analysis

To characterize *A. abdominalis* feeding behavior under no-choice conditions, functional response curves were fitted separately for each aphid clone × BSS treatment using the Holling Type II functional response model fitted by nonlinear least-squares regression (nlsLM, minpack.lm) assuming a 48 h experimental period [50]. Mean prey consumption (±SE) at each initial prey density was plotted together with the fitted curves. Because the functional response analysis was intended to provide a descriptive representation of predator feeding behavior, statistical inference regarding treatment effects was based on the generalized linear models described above.

#### 2.5.2. Hind Tibia Lengths Analysis

*Aphelinus abdominalis* adults’ hind tibia lengths (µm) were analyzed using one-way ANOVA, with treatment clones +5, −5, +7, and −7 as the fixed factor. Significant differences among treatment means were determined using Tukey’s HSD multiple-comparison test. Data are presented as means ± SE.

All statistical analyses were performed in R version 4.5.1 (R Core Team, Vienna, Austria) using RStudio Desktop: Version 2025.05.1+513 (Posit Software, PBC, Boston, MA, USA), and statistical significance was accepted at *p* < 0.05 [51].

## 3. Results

### 3.1. Elimination of Bacterial Secondary Symbionts (BSS) from Aphid Clones

**Figure 1:**
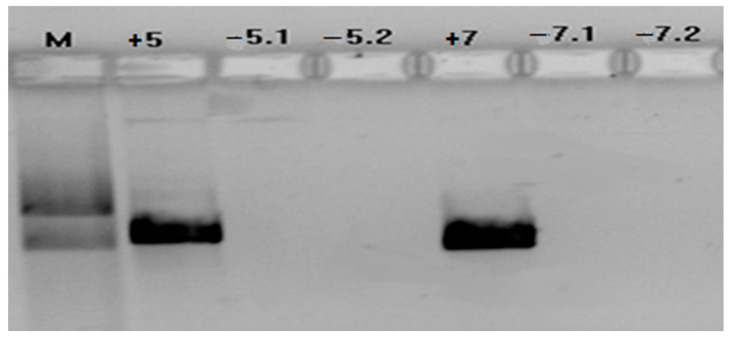
Diagnostic PCR confirmation of BSS elimination from wheat aphid clones.

Elimination of BSS in wheat aphids was confirmed using the diagnostic PCR. Antibiotic treatments resulted in four wheat aphid lines: clone 5 (HD) with BSS (+5), clone 5 without BSS (−5), clone 7 (RI) with BSS (+7), and clone 7 without BSS (−7) (Figure 1).

### 3.2. Effects of Bacterial Secondary Symbionts (BSS) on Host Feeding by Aphelinus abdominalis in No-Choice Assays

**Figure 2:**
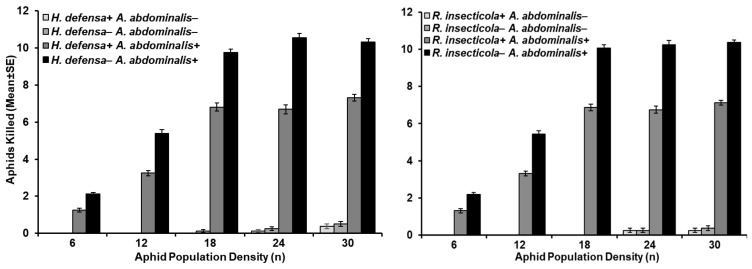
Effects of bacterial secondary symbionts (BSS) on wheat aphid host feeding by *Aphelinus abdominalis* under various host densities (no-choice assays).

Results from the no-choice assays involving clone 5 (*HD*) and clone 7 (*RI*) revealed that host killing by *A. abdominalis* in BSS-infected wheat aphids was less relative to BSS-free wheat aphids. At the lowest density (6 aphids), mean host mortality ranged from 1.25 (with BSS) to 2.12 (without BSS) in clone 5 and from 1.31 to 2.18 in clone 7. At the highest aphid density (30 aphids), mortality increased to 7.31 (BSS-infected) and 10.31 (BSS-free) in clone 5, and to 7.12 and 10.37, respectively, in clone 7. Control mortality was negligible at lower densities (6–18 aphids) and remained minimal at higher densities (Figure 2). The generalized linear model revealed that BSS status significantly influenced parasitoid-induced mortality (LR χ^2^ = 132.07, df = 1, *p* < 0.001). Aphids harboring BSS consistently experienced lower mortality than BSS-free aphids, indicating that BSS infection substantially reduced host susceptibility to natural-enemy attack. Host density also had a significant effect on mortality (LR χ^2^ = 103.15, df = 4, *p* < 0.001), demonstrating that predator performance varied across aphid densities. In contrast, the aphid clone had no significant effect on mortality (LR χ^2^ = 0.01, df = 1, *p* = 0.910), indicating that the overall response of the natural enemy was comparable between the two aphid clones (Table 1). After accounting for BSS status and host density, the estimated mortality probability was 15.1% for Clone 5 (HD) and 20.5% for Clone 7 (RI), with the odds of mortality being 1.45 times higher for Clone 7 than for Clone 5 (Table 2). Overall, host-feeding activity increased with host density but approached a plateau at higher densities, indicating a saturating functional response. No significant interaction between BSS status and host density was detected for host-killing behavior in these assays.

**Figure 3:**
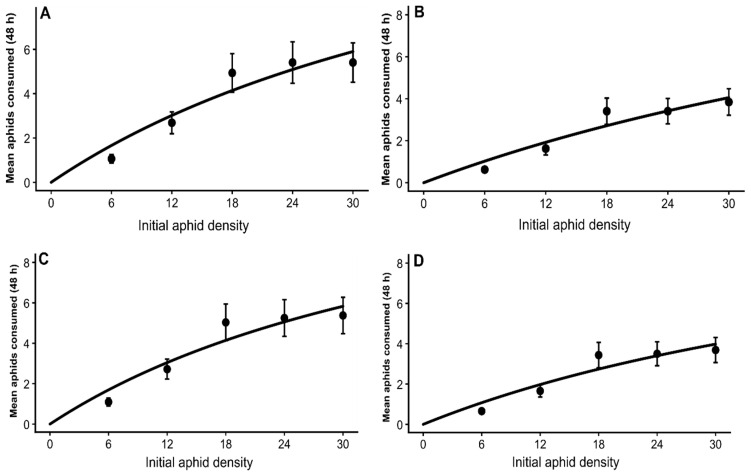
Functional response of *A. abdominalis* to aphid clones differing in bacterial secondary symbiont (BSS) status over a 48 h experimental period. Points represent the mean number of aphids consumed (±SE) at each initial prey density (n = 16 replicate arenas per density), and solid lines represent the fitted Holling Type II functional response models. Panels show (**A**) Clone 5 (BSS-free), (**B**) Clone 5 (BSS-infected); (**C**) Clone 7 (BSS-free), and (**D**) Clone 7 (BSS-infected).

Regarding the functional response of *A. abdominalis* to aphid clone and BSS status, *A. abdominalis* consumption increased with increasing initial aphid density under all treatment combinations and exhibited a saturating (Type II) functional response. Across both aphid clones, *A. abdominalis* consistently consumed more BSS-free aphids than BSS-infected aphids over the range of prey densities tested. In contrast, the overall shape and magnitude of the functional response were similar between Clone 5 (HD) and Clone 7 (RI), indicating that the aphid clone had little influence on predator feeding behavior, whereas the presence of BSS reduced prey consumption. These graphical patterns are consistent with the generalized linear model results, which identified BSS status and prey density as significant determinants of predation, whereas aphid clone had no significant effect (Figure 3).

### 3.3. Bacterial Secondary Symbionts (BSS) Mediated Host Preference in Choice Assays (BSS)

**Figure 4:**
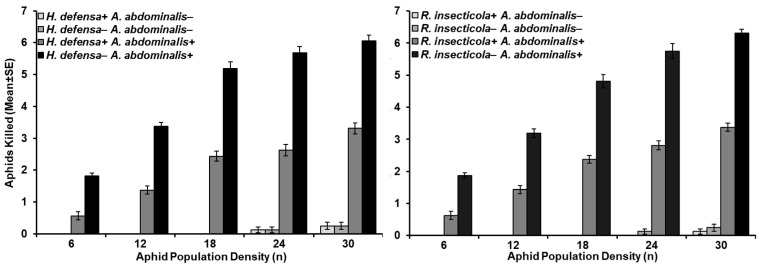
Effects of bacterial secondary symbionts (BSS) on wheat aphid host feeding by *Aphelinus abdominalis* under various host densities (BSS choice assays).

In this choice assay, *A. abdominalis* differentiated between BSS-infected and BSS-free aphids. Aphid mortality was significantly greater in BSS-free aphid individuals, whereas BSS-infected aphids experienced significantly lower mortality (*p* < 0.005; Figure 4).

In this dual-choice assay, neither aphid clone (LR χ^2^ = 0.08, df = 1, *p* = 0.773) nor host density (LR χ^2^ = 3.80, df = 4, *p* = 0.434) significantly affected *A. abdominalis* preference for BSS-free versus BSS-infected aphids. These findings indicate that the *A. abdominalis* exhibited a consistent BSS-free host-choice pattern across both aphid clones and throughout the range of host densities tested (Table 3). Also, model-based estimates demonstrated a clear overall preference for BSS-free aphids when both host types (BSS-free and BSS-infected) were simultaneously available. Across all aphid clones and host densities, the predator allocated 69.3% (95% CI: 66.0–72.5%) of successful attacks to BSS-free aphids, which was significantly greater than the expected equal allocation between the two host types (z = 10.45, *p* < 0.001; Table 4). This consistent preference indicates that BSS reduced the probability of aphids being selected and successfully attacked during direct host-choice encounters.

These results demonstrate that *A. abdominalis* preferentially allocated host-feeding events to BSS-free aphids when both host types were simultaneously available. Host-feeding mortality increased significantly with increasing aphid density, indicating greater predator feeding activity at higher host densities, whereas control mortality remained negligible. The interaction between BSS status and host density did not significantly improve model fit and was therefore excluded from the final generalized linear model. Accordingly, there was no statistical evidence that the parasitoid’s preference for BSS-free aphids varied across the range of host densities examined.

### 3.4. Effect of Clonal Variation in Host Preference in Choice Assays

**Figure 5:**
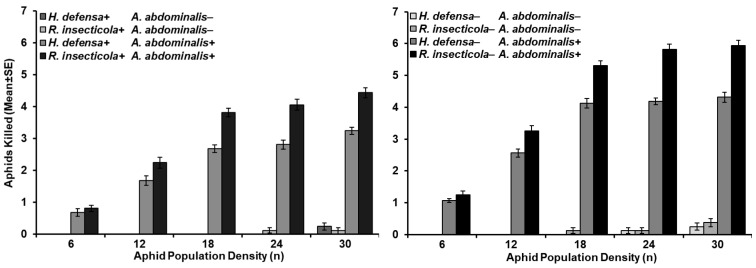
Effects of host clonal variation (Clone +5 and +7 and Clone −5 and −7) on wheat aphid host feeding by *Aphelinus abdominalis* under various host densities (clonal choice assays).

To evaluate whether aphid genetic background influenced host-feeding behavior, additional dual-choice assays were conducted between Clone 5 and Clone 7 carrying the same BSS status (+5 vs. +7 and −5 vs. −7). In both comparisons, *A. abdominalis* consistently allocated more host-feeding events to Clone 7 than to Clone 5 (Figure 5). This pattern was observed in both BSS-infected (+5 vs. +7) and BSS-free (−5 vs. −7) aphids, indicating that the genetic difference persisted after removal of BSS. The persistence of this pattern following the removal of BSS suggests that host genetic background may contribute to differences in susceptibility to host feeding. However, because the naturally infected clones originally carried different BSS species, comparisons between the infected clones alone cannot distinguish genotype effects from symbiont species effects. Consequently, these findings should be regarded as preliminary and interpreted with caution. In the mixed-clone dual-choice assay, neither bacterial secondary symbiont (BSS) status (LR χ^2^ = 0.10, df = 1, *p* = 0.756) nor host density (LR χ^2^ = 0.49, df = 4, *p* = 0.974) significantly influenced predator allocation between Clone 5 (HD) and Clone 7 (RI) (Table 5). These findings indicate that predator preference between the two aphid clones remained consistent regardless of BSS status or prey density. Model-estimated probabilities showed that 43.1% (95% CI: 39.5–46.7%) of realized predator attacks were allocated to Clone 5 (HD), which was significantly lower than the null expectation of equal allocation between the two aphid clones (z = −3.71, *p* < 0.001). Consequently, approximately 56.9% of predator attacks were directed towards Clone 7 (RI), indicating a modest but consistent overall preference for Clone 7 across all experimental conditions (Table 6).

### 3.5. Effect of Bacterial Secondary Symbionts (BSS) on A. abdominalis Adult Body Size

**Figure 6:**
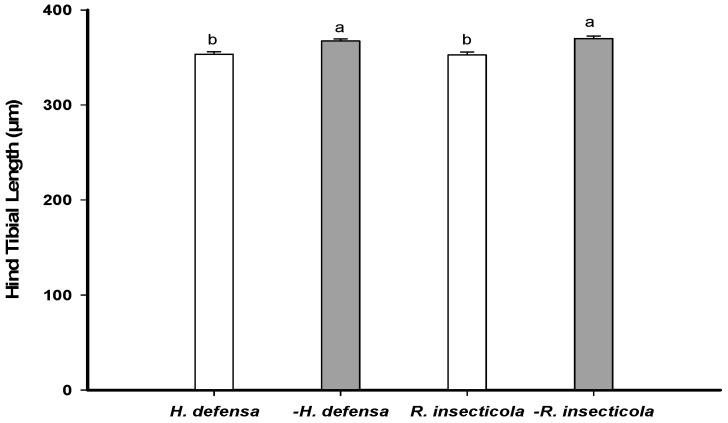
Hind tibia length of *Aphelinus abdominalis* developed in *Sitobion avenae* (BSS-infected and BSS-free). Bars represent mean ± SE based on 30 adult parasitoids per treatment. Different letters above the bars indicate significant differences according to one-way ANOVA followed by Tukey’s HSD multiple-comparison test (*p* < 0.05).

The hind tibia length (for adult body size estimation) differed significantly among treatments (F_3_,_116_ = 6.154, *p* = 0.00064; Figure 6). Adult *A. abdominalis* emerging from BSS-free aphids were significantly larger than those emerging from BSS-infected aphids. In Clone 7, the mean hind tibia length was 369.92 ± 3.56 µm (mean ± SE) in parasitoids emerging from BSS-free aphids (−7), compared with 352.64 ± 3.25 µm in parasitoids emerging from BSS-infected aphids (+7). Likewise, parasitoids emerging from Clone −5 had a mean hind tibia length of 367.36 ± 3.88 µm, whereas those emerging from Clone +5 averaged 353.28 ± 3.97 µm. Tukey’s HSD test showed that the two BSS-free treatments did not differ significantly from one another, nor did the two BSS-infected treatments; however, each BSS-free treatment produced significantly larger parasitoids than its corresponding BSS-infected treatment (*p* < 0.05). These findings indicate that BSS exerted sublethal effects on adult parasitoid body size.

### 3.6. Transmission of Bacterial Secondary Symbionts Through Aphelinus abdominalis

DNA was extracted from *A. abdominalis* females recovered after exposure to BSS-infected aphids and from adult parasitoids emerging from BSS-infected aphid mummies. Diagnostic PCR did not detect bacterial secondary symbionts (BSS) in any parasitoid samples analyzed. Under the laboratory conditions of this study, these results indicate that *A. abdominalis* did not acquire detectable BSS from infected aphid hosts, and no evidence was found that the parasitoid mediated horizontal transmission of BSS. However, these findings are limited to the sensitivity of the qualitative diagnostic PCR assay and the experimental conditions employed.

## 4. Discussion

Bacterial secondary symbionts (BSS) persist within aphid populations only if they confer measurable fitness benefits to their hosts. Numerous studies have demonstrated that natural enemies are inhibited by certain BSS, including parasitoids and pathogens [8,37,38,39,52]. For instance, *H. defensa* is known to reduce parasitism success in aphids [53]. Our findings extend this body of work by showing that BSS also reduce host mortality caused by parasitoid host feeding, a dimension that has received little prior attention. Across all host densities, aphids harboring BSS experienced consistently lower mortality than symbiont-free individuals. This pattern suggests that BSS-mediated protection is not limited to parasitism but also affects non-reproductive host-killing behaviors. Given the absence of prior studies explicitly addressing host-feeding resistance mediated by BSS, our results provide new evidence that these symbionts broaden their protective role within aphid–parasitoid interactions. Such multifunctional protection may contribute to the persistence and spread of BSS in aphid populations. From an applied perspective, this finding raises concerns for biological control programs, as symbiont-mediated resistance could reduce the overall effectiveness of parasitoids against pest aphids. One shortcoming of the study is that we do not have antibiotic-treated control lines still carrying their endosymbionts during the generation of BSS-free aphid lines through antibiotic curing. Although the protocols used for the curing have already been documented, and the successful removal of BSS was confirmed via PCR for as many as eight generations, we cannot rule out completely the possibility of side effects caused by antibiotics, affecting the physiology of aphids as well as some parts of the microbial community apart from BSS removal. While the findings are consistent with the hypothesis that BSS can protect aphids, some unknown side effects stemming from the curing procedures cannot be excluded. The future studies that rely on sham-injected control lines or different methods to test the symbiont status of the insects will allow drawing even more reliable conclusions about the BSS protective effect.

Parasitoid foraging behavior typically exhibits a positive relationship with host density for both parasitism and host feeding [54,55,56,57,58,59]. Our results for *A. abdominalis* are consistent with this general pattern. Host-feeding mortality increased with increasing aphid density, and the fitted Holling Type II functional response curves indicated that prey consumption approached an asymptote at higher host densities. Across all densities, BSS-infected aphids consistently experienced lower host-feeding mortality than BSS-free aphids. The generalized linear model further showed significant effects of BSS status and host density on host-feeding mortality, whereas no statistical evidence was found that the effect of BSS varied across the host-density range examined. These findings suggest that BSS reduced overall host-feeding mortality without appreciably altering the overall pattern of the predator’s density-dependent feeding response. Choice experiments revealed that BSS influenced the host-feeding behavior of *A. abdominalis*. When simultaneously presented with BSS-infected and BSS-free aphids, parasitoids preferentially fed on symbiont-free hosts. These results demonstrate that parasitoids responded differently to BSS-infected and BSS-free aphids, although the specific cues underlying this behavioral response were not investigated in the present study. Previous work has shown that parasitoids can respond to symbiont-associated host characteristics [35], although most studies have focused on parasitism rather than host feeding. Cheng et al. reported parasitoid preference in relation to the primary symbiont *Buchnera aphidicola*, whereas comparable evidence for bacterial secondary symbionts has been limited [60]. Within each aphid clone, the BSS-infected and BSS-free lines (+5 vs. −5 and +7 vs. −7) were genetically identical except for BSS status; the observed differences in host-feeding behavior are consistent with an effect of BSS status on host acceptance or suitability. However, further studies are required to identify the behavioral or physiological cues responsible for this response. In addition to the effects of bacterial secondary symbionts, differences in host-feeding susceptibility were observed between the two aphid clones examined. Across both BSS-infected and BSS-free treatments, Clone 5 was consistently less susceptible to host feeding than Clone 7. Because this pattern persisted following the removal of BSS, it suggests that host genetic background may contribute to variation in susceptibility. Previous studies have likewise reported clone-dependent variation in aphid susceptibility to parasitoids. For example, Li et al. [61] demonstrated differences among pea aphid clones in resistance to *Aphidius ervi*, whereas von Burg et al. [36] reported comparable genotype-dependent responses in *Myzus persicae*. Similarly, Ferrari et al. [62] and Vorburger et al. [37] highlighted the importance of host genetic variation in aphid–parasitoid interactions. However, because only two naturally occurring aphid clones were included in the present study, these findings should be regarded as preliminary and should not be generalized to *S. avenae* populations as a whole. Future studies incorporating a larger number of aphid genotypes will be required to determine the broader contribution of host genetic variation to host-feeding susceptibility. The combined effects of host genotype and BSS are nevertheless likely to contribute to variation in parasitoid performance under natural conditions. Choice assays of the current study use the hind tarsus clipping for tagging aphid groups. Though the clipped method was switched between BSS-infected and BSS-free aphid groups to avoid any systematic error. It was not checked if this marking method influenced aphids’ behavior or the parasitoids’ selection of hosts. Accordingly, we cannot fully discard the possibility of the minor effect of marking here. Future studies using non-invasive marking methods might give an opportunity for validation of these findings without being influenced by physical marking.

Previous studies have shown that symbionts such as *H. defensa* and *R. insecticola* can reduce parasitoid growth and prolong development [13,14]. Our results support these observations. Parasitoids emerging from BSS-infected aphids exhibited significantly shorter hind tibiae, indicating reduced adult body size. Although the present study did not directly measure fitness-related traits such as fecundity, longevity, or mating success, adult body size is widely recognized as an important predictor of these characteristics in many parasitoid species. Therefore, the observed reduced body size may have ecological consequences by potentially influencing parasitoid performance. The successful emergence of parasitoids from BSS-infected hosts indicates that BSS did not completely prevent parasitoid development; however, the reduction in adult body size suggests that BSS exerts sublethal effects on parasitoids. Consequently, even when parasitoid development is successful, bacterial secondary symbionts may negatively influence higher trophic levels through reductions in adult body size.

BSS are primarily transmitted vertically through host reproduction, although occasional horizontal transmission has been documented [15,17,18]. Proposed mechanisms include sexual transfer, ingestion, and transfer via parasitoids [16,18,63]. In the present study, diagnostic PCR did not detect BSS in *A. abdominalis* individuals, either after host exposure or following emergence from BSS-infected aphids. Under the laboratory conditions and sampling intervals employed, these findings provide no evidence that *A. abdominalis* acquired detectable BSS or mediated detectable horizontal transmission of these symbionts. This finding agrees with earlier studies indicating that parasitoids are unlikely to act as efficient vectors for the transmission of BSS [15]. Nonetheless, as the detection was performed using a qualitative diagnostic PCR assay and the experiment included a single parasitoid species in the laboratory, any transmission below the detection threshold or under different environmental conditions is still a possibility. In evolutionary terms, the fact that there is limited parasitoid-mediated horizontal transmission could be favorable because acquiring symbionts could potentially decrease the fitness of the parasitoid.

## 5. Conclusions

This study indicates that bacterial secondary symbionts (BSS) influence aphid–parasitoid interactions by reducing host-feeding mortality, altering host-feeding responses, and decreasing the adult body size of *A. abdominalis* under laboratory conditions. These responses may influence parasitoid performance and therefore have potential implications for the biological control of *S. avenae*. Additionally, differences among the clones suggest that clonal background may also contribute to variations in aphid–parasitoid interactions; however, additional studies employing reciprocal symbiont transfers and multiple aphid genotypes are needed to distinguish host genotype effects from other clone-associated factors. Together, these findings highlight the complexity of multitrophic interactions and underscore the importance of considering both symbiont-mediated and host genetics while evaluating the biological control strategies against aphid pests.

## Figures and Tables

**Table 1:** Effects of aphid clone, bacterial secondary symbiont (BSS) status and host density on *Aphelinus abdominalis*-induced mortality in the no-choice assay.

Source of Variation	df	LR χ^2^	*p*-Value
Aphid clones	1	0.013	0.910
BSS status	1	132.065	<0.001 *
Host density	4	103.145	<0.001 *

Values are from Type III likelihood-ratio (LR) χ^2^ tests of the final additive binomial GLM. Asterisks indicate statistical significance: * *p* < 0.001.

**Table 2:** Model-estimated *Aphelinus abdominalis* preference for BSS-free aphids in the no-choice assay.

Parameter	Estimate
Mortality probability (Clone 5; HD)	0.151
Mortality probability (Clone 7; RI)	0.205
*p*-value	<0.001

Estimated marginal means were obtained from the final additive binomial GLM after adjusting for bacterial secondary symbiont (BSS) status and host density. Odds ratios are presented on the response scale.

**Table 3:** Effects of Bacterial Secondary Symbionts (BSS) and host density on *Aphelinus abdominalis* preference for BSS-free versus BSS-infected aphids in the dual-choice assay (BSS).

Source of Variation	df	LR χ^2^	*p*-Value
Aphid clone	1	0.083	0.773
Host density	4	3.798	0.434

Values are from Type III likelihood-ratio (LR) χ^2^ tests of the final additive binomial GLM. *A. abdominalis* preference was analyzed as an allocation of its attacks between BSS-free and BSS-infected aphids.

**Table 4:** Model-estimated *Aphelinus abdominalis* preference for BSS-free aphids in the dual-choice assay (BSS).

Parameter	Estimate
Probability of allocation of kills to BSS-free aphids	0.693
95% Confidence interval	0.660–0.725
Test against equal choice (0.50)	z = 10.45
*p*-value	<0.001

Values from Type III likelihood-ratio (LR) χ^2^ tests of final additive binomial generalized linear model (GLM). *A. abdominalis* preference was analyzed as the allocation of predator attacks between BSS-free and BSS-infected aphids.

**Table 5:** Effects of clonal variation and host density on *Aphelinus abdominalis* host preference between aphid clones in the mixed-clone dual-choice assay.

Source	df	LR χ^2^	*p*
BSS status	1	0.096	0.756
Host density	4	0.491	0.974

Values are from Type III likelihood-ratio (LR) χ^2^ tests of the final additive binomial GLM.

**Table 6:** Model-estimated allocation of *Aphelinus abdominalis* host killing between Clone 5 (HD) and Clone 7 (RI) in the mixed-clone dual-choice assay.

Parameter	Estimate
Probability that a realized kill was allocated to Clone 5 (HD)	0.431
95% confidence interval	0.395–0.467
Test against equal allocation (0.50)	z = −3.71
*p*-value	< 0.001

Values are derived from estimated marginal means of the final additive binomial generalized linear model (GLM). The estimated probability represents the proportion of *A. abdominalis* attacks allocated to Clone 5 (HD) after averaging across bacterial secondary symbiont (BSS) treatments and host densities. A probability of 0.50 represents equal allocation of *A. abdominalis* attacks between the two aphid clones.

## Data Availability

All the data sets are presented in the manuscript.
